# Inhibitory Effect of Bovine Lactoferrin on Catechol-*O*-Methyltransferase

**DOI:** 10.3390/molecules22081373

**Published:** 2017-08-19

**Authors:** Masayuki Ikeda, Hiroshi Iijima, Ichizo Shinoda, Hiroshi Iwamoto, Yasuhiro Takeda

**Affiliations:** 1 Wellness & Nutrition Science Institute, R&D Division, Morinaga Milk Industry Co., Ltd., Zama, Kanagawa 252-8583, Japan; i_sinoda@morinagamilk.co.jp (I.S.); h_iwamot@morinagamilk.co.jp (H.I.); ya_taked@morinagamilk.co.jp (Y.T.); 2 School of Pharmacy, Nihon University, Funabashi, Chiba 274-8555, Japan; iijima.hiroshi@nihon-u.ac.jp

**Keywords:** enzyme inhibitor, multifunctional protein, lactoferrin, catechol-*O*-methyltransferase

## Abstract

Lactoferrin (LF) is a well-known multifunctional protein. In this study, we report the inhibitory potency of bovine LF (bLF) on catechol-*O*-methyltransferase (COMT), which catalyzes methylation of catechol substrates. We found that bLF binds to and inhibits COMT using its *N*-terminal region. An *N*-terminal peptide fragment obtained from bLF by trypsin digestion showed a higher inhibitory activity than intact bLF. A synthetic fragment of the bLF *N*-terminal residues 6–50, with two pairs of disulfide bonds, also showed higher inhibitory activity than intact bLF. Enzyme kinetic studies proved that bLF did not compete with *S*-adenosylmethionine (the methyl donor substrate) as well as methyl acceptor substrates such as dihydroxybenzoic acid, (−)-epicatechin, norepinephrine, or l-3,4-dihydroxyphenylalanine. The inhibitory potency of bLF decreased against a COMT preparation pretreated with dithiothreitol, suggesting that the oxidation status of COMT is relevant to interaction with bLF. We further confirmed that COMT activity in the cell extracts form Caco-2 and HepG2 cells was inhibited by bLF and by the synthesized fragment. Enzyme kinetic study indicated that bLF functions as a non-competitive inhibitor by binding to an allosteric surface of COMT.

## 1. Introduction

Lactoferrin (LF) is a multifunctional protein distributed in body fluids such as milk and mucus, in addition to secondary granules of white blood cells [1,2,3]. Its functions include immunomodulation [4,5,6] and regulation of gastrointestinal flora [7]. Moreover, LF is a ferric-binding protein and shows enhanced antibacterial activity by chelating ferric ions. Its *N*-terminal peptide, which is produced by pepsin digestion, is known as lactoferricin B (LFcinB) and shows antibacterial activity [8]. LF binds to proteins such as intelectin [9], low-density lipoprotein receptor-related protein [10], nucleolin [11], Glyceraldehyde-3-phosphate dehydrogenase (GAPDH) [12], calmodulin [13], and lipopolysaccharide [14]. These properties may facilitate LF’s multifunctionality, and because it is a milk protein and is habitually ingested with other foods, its function is complex.

Cocoa and tea are known to have beneficial effects on human health with regard to vascular disease and metabolic syndrome. These plant-derived foods contain large amount of polyphenols that impact biological functions [15]. Catechins are representative polyphenols, many of which have a catechol substructure and have been studied regarding metabolism kinetics. Ingestion with milk has been discussed with respect to the bioavailability of catechins, and the interaction of milk and catechins has been investigated in clinical trials [16,17]. The direct interactions of milk proteins, such as casein with catechins, have also been discussed with respect to bioavailability of catechins [18,19]. However, the beneficial effects of simultaneous ingestion remain unclear. On the other hand, the bioavailability of absorbed catechols would be affected by various metabolic enzymes such as glycosidases and sulfatases. In particular, methylation of catechins by catechol-*O*-methyltransferase (EC 2.1.1.6) (COMT) is one of the major metabolic routes in vivo. As COMT inactivates catechol amine neurotransmitters, it has been investigated from the perspective of inhibitors and genetic background [20,21,22].

COMT is expressed in various organs, with high activity in the liver and kidneys [23,24]. It is also expressed in the gut, especially in the intestinal mucosa [25]. The physiological role of COMT in the metabolism of neurotransmitters has been a focus in the pharmaceutical sciences [26,27].

COMT inhibitors are medically significant. Inhibitors such as entacapone and tolcapone are used to manage symptoms of Parkinson’s disease by maintaining plasma concentrations of l-3,4-dihydroxyphenylalanine (L-DOPA) [28]. On the other hand, natural COMT inhibitors, such as epigallocatechin gallate and quercetin, are found in many types of foodstuffs [29,30]. However, high-molecular-weight inhibitors of COMT have not been widely studied, except for antibodies [31].

COMT activity is regulated by many factors, including pH, magnesium ions, tissue preparation, etc. [32]. COMT occurs as two molecular types, namely soluble COMT and membrane-bound COMT. The COMT molecule contains many thiol (SH) groups (seven cysteinyl residues in human COMT), and its activity is known to be influenced by the oxidation state [33].

COMT is expressed in the intestine, but it is an intracellular protein. In the intestine, cells are renewed continuously, and in this process, old cells are removed from the intestinal mucosa [34]. These old cells might leak intracellular proteins in neighboring areas. Actually, in intestinal areas, the activity of the intracellular enzyme lactate dehydrogenase (LDH) has been detected. In general, infection or mechanical damage to organs can trigger the leakage or secretion of intracellular materials, such as COMT [35].

bLF is ingested with milk, cheese, and supplements. After ingestion, bLF is exposed to gastric enzymes, and it might lose its activity. Therefore, gastric digestion of bLF was investigated in humans, and it was shown that intact bLF is present in the intestine [36,37].

During the course of our study to characterize bLF, we encountered the inhibitory potency of bLF on COMT activity. In this study, we discuss the site for the binding of bLF to COMT, the effect of bLF on substrate affinity, and the sensitivity of COMT to bLF in relation to its oxidation status.

## 2. Results

### 2.1. Interaction (Inhibitory) Sites of bLF with COMT

**Figure 1:**
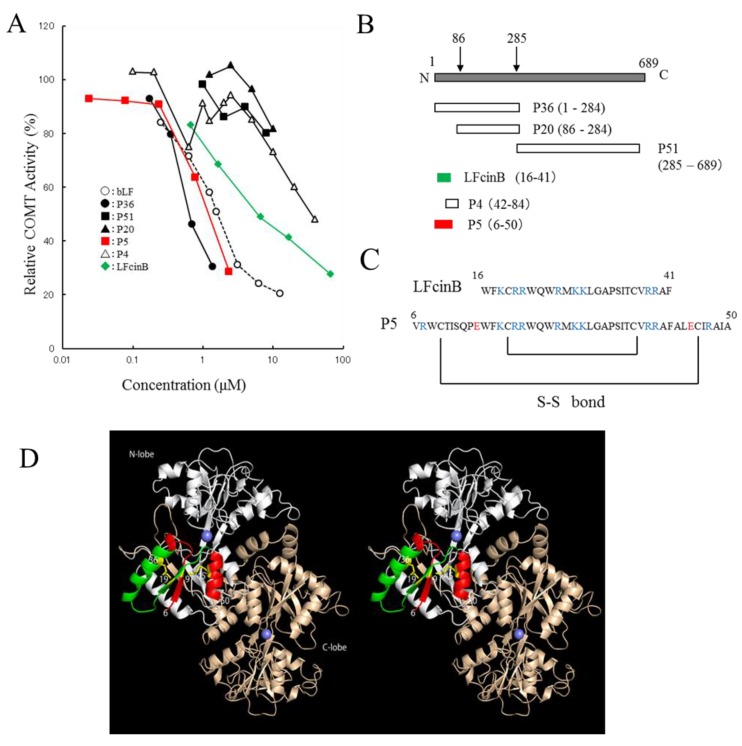
Inhibitory activity of bovine lactoferrin (bLF)-derived peptides on catechol-*O*-methyltransferase (COMT): (**A**) The horizontal axis represents peptide concentration. The vertical axis represents relative COMT activity in %. (**B**) The mapping of peptides. P36, P20, and P51 are trypsin-digested fragments derived from bLF. Their *N*-terminal amino acid sequences were analyzed and their molecular weights were estimated by SDS-PAGE analysis. The sequence and molecular weights determined their mapping as presented in this panel. (**C**) Amino acid sequence of LFcinB and P5. Basic amino acids (K, R) are shown in blue, and acidic amino acids (E) are shown in red. The two disulfide bonds are conserved in native bLF. (**D**) Stereo view (wall-eye) representation of bLF (Protein Data Bank entry 1BLF [39]). P5 (6–50) region: red; LFcinB (16–41) region: green; C-lobe (P51 285–689) region: light brown. Disulfide bridges (Cys9–Cys45 and Cys19–Cys36) are shown.

bLF exhibited higher inhibitory potency on COMT activity than did other milk-derived proteins, with an IC_50_ value of 1.6 µM (Table S1). Trypsin-digested peptide fragment P36 (1–284) indicated inhibitory activity, whereas P20 (86–689) and P51 (285–689) showed no inhibition (Figure 1A,B). Based on this result, P5 (6–50) and P4 (42–84) were chemically synthesized (Figure 1C). P36 and P5 were more active than intact bLF, whereas LFcinB was less active than intact bLF. A remarkable difference in the inhibitory potency of P5 and P4 was observed. The sequence of P5 (6–50) includes the LFcinB region (16–41). However, LFcinB is less active than P5. P5 reserves the two disulfide bonds (Cys9–Cys45 and Cys19–Cys36) whereas LFcinB keeps only one disulfide bond. Thus, the interaction site of bLF is located in the P5 region and existence of the two sulfide bonds is important. These disulfide bonds should constrain the flexibility of P5 and stabilize the active conformation that interacts with COMT. In the crystal structure of buffalo LF (Protein Data Bank (PDBj) Japan entries 1BIY [38] and 1BLF [39]), the P5 region is located on the molecular surface of LF and forms a β1/α1/β2/α2 type compact lobe, where α and β represent α-helix and β-sheet secondary structures. It should be noted that when the disulfide bonds were absent, P5 was insoluble in water (data not shown).

### 2.2. Enzyme Kinetic Analysis: Inhibition of COMT by bLF

**Figure 2:**
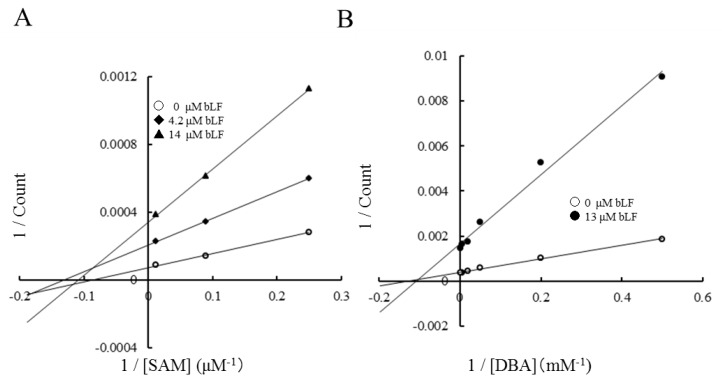
Enzyme kinetic analysis of the inhibitory activity of bLF on COMT activity: (**A**) Lineweaver–Burk plot of the COMT reaction with varying concentrations of *S*-adenosylmethionine (SAM) (4, 11.2, and 83.2 μM). *K*_m_^SAM^ was calculated from the intercepts of the horizontal axis to be 11.4, 8.0, and 10.3 μM. (**B**) Lineweaver–Burk plot of the COMT reaction with varying concentrations of dihydroxybenzoic acid (DBA) (2, 5, 20, 50, 200, and 500 μM). The concentrations of bLF in the reaction mixture are given in the graph legend.

The inhibitory mechanism of bLF on the COMT reaction was analyzed by an enzyme kinetic study. The presence of bLF affected only the *V*_max_ of the reaction. As shown in the Lineweaver–Burk plot (Figure 2), the apparent Michaelis constants *K*_m_ for *S*-adenosylmethionine (SAM) and dihydroxybenzoic acid (DBA) were not affected by the presence of bLF. *K*_m_^SAM^ was 8–11 µM (Figure 2A) and *K*_m_^DBA^ was 110–130 µM (Figure 2B). These facts suggest that bLF does not compete with the methyl donor substrate (SAM) and methyl acceptor substrate (DAB). Considering that those substrates are small molecules and the substrate-binding sites are located inside of the enzyme, COMT should interact with bLF at an allosteric molecular surface rather than at substrate-binding sites. This inhibitory activity of bLF was consistently observed when epicatechin, L-DOPA, or norepinephrine was employed as the methyl acceptor substrate (Figure S1).

### 2.3. Inhibitory Potency of bLF on dithiothreitol (DTT)-Treated COMT

**Figure 3:**
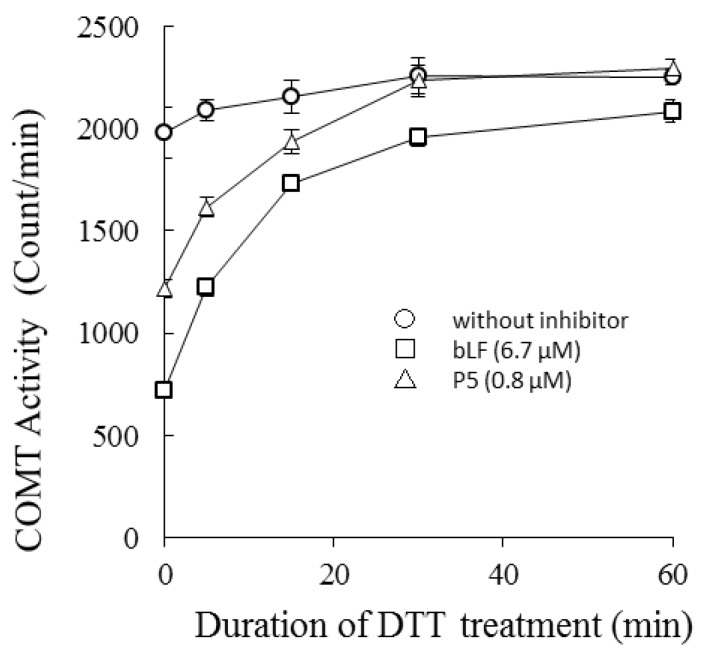
Effects of bLF and P5 on dithiothreitol DTT)-treated COMT (time course experiments): COMT was treated with 1.2 mM DTT for 0–60 min at 37 °C, and then the catalytic activity of the treated preparations was measured in the presence or absence of bLF or P5 (DTT final concentration: 1.0 mM). The horizontal axis represents the duration of DTT treatment of COMT in minutes. The vertical axis represents the catalytic activity of COMT expressed by the amount of methyl transfer (scintillation counts). This experiment was performed with triplicate measurements and values were described as average and standard deviation (SD).

COMT was treated with 1.2 mM DTT for 0–60 min at 37 °C, and then the catalytic activity of the treated preparations were measured in the presence or absence of bLF or P5 (Figure 3). Susceptibility of each COMT preparation to bLF inhibition was dependent on incubation time with DTT. Longer treatments led to reduced inhibitory activity, i.e., the inhibition potency of bLF was lowered and the reaction velocity recovered. The binding potency of bLF to COMT with DTT treatment was assessed by ELISA (Figure S2). DTT treatment of COMT lowered the binding affinity between bLF and COMT.

### 2.4. Effects of bLF on COMT Activity in Lysates from Human Cell Lines

**Figure 4:**
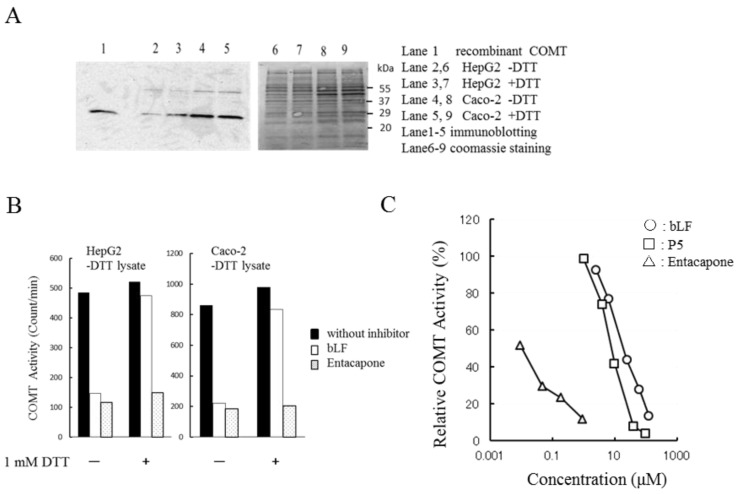
Effects of bLF and P5 on COMT activity in cell lysates: (**A**) Immunostaining of COMT (lanes 2–5) and Coomassie staining (lanes 6–9) of protein extracted from HepG2 and Caco-2 cells. Lanes 2–9 were loaded with equal volumes of cell lysates. Loaded proteins: 15 μg (HepG2), 20 μg (Caco-2 −DTT), and 22 μg (Caco-2 +DTT). Lane 1 is recombinant human soluble COMT. Lysates designated as +DTT are lysates prepared using extraction buffer containing 5 mM DTT. Those designated as −DTT are lysates prepared in the absence of DTT; (**B**) Susceptibility of −DTT lysates to inhibitors. Effect of bLF and entacapone to COMT activity (−DTT lysates) was investigated in the reaction mixtures that contained 1 mM DTT or did not. Concentrations of bLF and entacapone were 63 μM and 0.19 μM, respectively. The vertical axis represents COMT activity expressed as counts per minute; (**C**) Concentration dependency of COMT activity on inhibitors. The horizontal axis represents the concentration of inhibitors. COMT activity of the −DTT lysate prepared from Caco-2 cell was examined. The reaction was performed in the absence of DTT.

Most experiments in this study were performed using recombinant human soluble COMT. To confirm the inhibitory potency of bLF on native COMT, we prepared cell lysates from human cell lines. Figure 4A shows SDS-PAGE of cell lysates prepared from HepG2 cells and Caco-2 cells. No evident difference was found between the +DTT and −DTT preparations. Immunostaining (panel A) shows that all lysates contained the soluble type COMT (MW 25 KDa). COMT activities between the +DTT and −DTT preparations were comparable (Figure S3). COMT activities of −DTT lysates were measured in the presence or absence of DTT in the reaction mixture (Figure 4B). Lysates from HepG2 cells and Caco-2 cells showed equivalent COMT activity regardless of the presence or absence of DTT in the reaction mixture. The inhibitory effect of bLF on COMT was lower in the presence of DTT (1 mM) than in the absence of DTT. In contrast, the inhibitory efficacy of entacapone was constant regardless of the presence or absence of DTT (Figure 4B). The inhibitory potency of P5 and bLF observed for recombinant COMT (Figure 1A) was reproduced in the native enzyme preparations (Figure 4C).

## 3. Discussion

Inhibition tests using trypsin-digested fragments and synthetic peptides indicated that bLF binds to COMT with its *N*-terminal region (Figure 1D). The crystal structure of bLF shows that it consists of *N*- and *C*-terminal lobes [38]. The sequence of P36 (1–284) covers most of the *N*-terminal lobe, whereas P51 (285–689) covers the *C*-terminal lobe. bLF interacts with COMT by the *N*-terminal lobe. P20 (86–284) did not indicate inhibitory potency, whereas P5 (6–50) indicated remarkable potency, suggesting that the latter half of the *N*-terminal lobe is not involved in the interaction with COMT. The sequence of P5 (6–50) includes that of LFcinB. The potency of peptides is in the order of P4 < LFcinB < bLF < P5 < P36 (Figure 1A). In the crystal structure, P5 makes a structural unit having a β1/α1/β2/α2 type fold (Figure 1D). LFcinB is a part of P5 and consists of an α1/β2 unit, which is presumably stabilized by a disulfide bond between Cys19 and Cys36. P5 is further stabilized by an additional disulfide bond between Cys9 and Cys45. Figure 1D maps the regions corresponding to P5 and LFcinB on the bLF structure. The P5 region would be the interface to COMT.

We found that heat or acid treatment of bLF enhanced its inhibitory activity (data not shown). We assumed that the P5 region would be able to maintain the β1/α1/β2/α2 fold in the denatured bLF due to conformational stability provided by the double disulfide bonds. The crystal structure of LFcinB has also been determined [40]. The conformation of LFcinB was found to be different from its conformation in the crystal structure of bLF. Indeed, the inhibitory potency of LFcinB is lower than that of P5. It is considered that the active conformation of the LFcinB region is stabilized by the second disulfide bond, making P5 a potent inhibitor.

The *N*-terminal region of bLF is rich in basic amino acid residues. The estimated isoelectric points of LFcinB and P5 calculated by ExPASy (SIB Bioinformatics Resource Portal) [41] are 11.8 and 10.8, respectively. bLF is known to interact with intelectin and LDL-related receptor [9,10]. For binding to these, charges in the *N*-terminal region of bLF are known to be important. The three-dimensional structure and charge distribution of the interaction site represented by P5 of bLF may be important for COMT inhibition.

COMT has two substrate-binding sites, one for the methyl donor substrate (SAM) and another for various methyl accepting substrates, such as catechol amine neurotransmitters (norepinephrine, epinephrine, and dopamine), 2-hydroxyestradiol, catechins, DBA, and others. Many small molecule inhibitors are known to bind to the methyl acceptor pocket of COMT [42]. Therefore, these inhibitors are considered competitive inhibitors.

As shown in Figure 2, Lineweaver–Burk plots indicated that inhibition by bLF is non-competitive. *K*_m_ values of methyl donor (SAM) and acceptor substrates (DBA) were constant, but *V*_max_ was reduced. These results clearly indicate that the binding site for bLF is independent from the methyl acceptor substrate site and methyl donor (SAM) site. Taken together, bLF binds to the allosteric site of COMT and affects the apparent reaction velocity. This means that in generation of the COMT/bLF/X complex, where X is null, SAM, SAM/methyl acceptor substrate, *S*-adenosylhomocysteine (SAH)/methylated substrate, or SAH, reduces the availability of functional COMT.

After a gentle treatment of COMT with 1.2 mM DTT at 37 °C for 60 min, treated COMT reduced its susceptibility to bLF. This indicates that COMT has a site that is readily reduced by DTT. Its reduction does not affect the catalytic ability of COMT but affects the binding affinity of COMT to bLF. The catalytic ability of COMT is known to decrease under states of oxidation [33]. This phenomenon was explained by modification of thiol cysteine residues. In our experiment, gentle treatment with DTT did not significantly affect catalytic ability, suggesting that the reduction site relevant for bLF binding is not involved in catalytic activity.

Cell lysates were prepared from HepG2 cells and Caco-2 cells using a buffer containing 5 mM DTT (+DTT lysate) or no DTT (−DTT lysate). Immunostaining of SDS-PAGE confirmed that COMT in cell lysates was the soluble type (Figure 4A). Sensitivity of the extracts (−DTT lysate) to entacapone inhibition was constant between −DTT and +DTT (1 mM). However, sensitivity to bLF inhibition was higher in the −DTT (Figure 4B). Inhibition by P5 was stronger than by bLF (Figure 4C). These results were all consistent with those found in experiments with recombinant enzymes.

**Figure 5:**
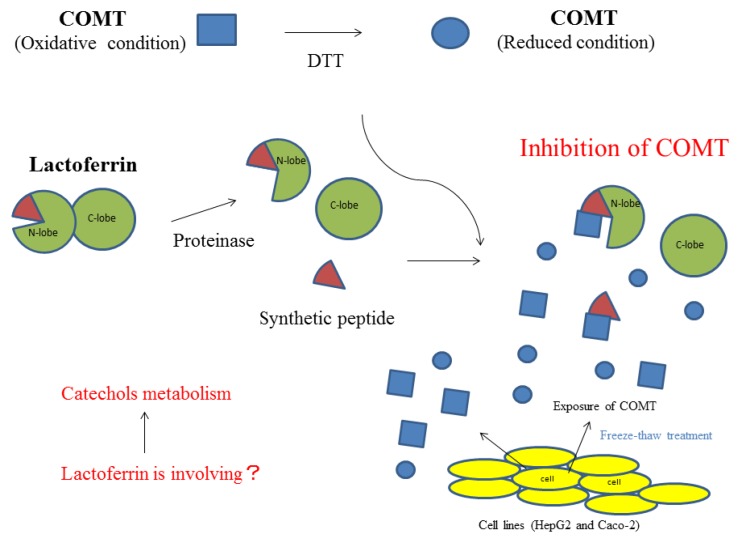
Summary of bLF inhibitory activity on COMT.

A summary of the inhibitory activity of bLF on COMT is shown in Figure 5. Higher order structural changes will enhance the inhibitory activity of bLF. Moreover, it will recognize the oxidative state of COMT and preferably inhibit COMT that is in an oxidized state.

Human LF has a basic region of amino acid sequences in the *N*-terminal region, which also has two disulfide bonds, and it can inhibit COMT activity especially after heat denaturation (data not shown). On the other hand, apo-transferrin, which also has two disulfide bonds in the *N*-terminal region, has no inhibitory activity (Table S1). The sequences and the three-dimensional structures of LF and transferrin are known to be conserved among many animal species. The reason for the difference in COMT inhibitory activity between LF and transferrin requires further investigation.

In the case of organ damage involving mechanical injury or infection, cells might undergo necrosis and intracellular materials might be released to the surrounding space. COMT is one such material that can affect catecholamine metabolism. COMT might augment inflammation and infection by influencing vascular function. LF is believed to maintain the microenvironment in the injury region through mucosal fluids or neutrophils that contain LF. To confirm this speculation, further experiments are needed.

LF exists in body fluids and neutrophils. The concentration of human LF is 1.6–2.1 mg/mL in milk and 1.73 mg/mL in tears [43,44], and that of bLF is 0.031–0.485 mg/mL in milk [45]. These concentrations are sufficiently high to inhibit COMT. The results of this study suggest that LF functions to regulate COMT activity and that it influences the metabolism of exogenous and endogenous catechol substrates in vivo.

## 4. Materials and Methods

### 4.1. Chemicals

bLF was produced by Morinaga Milk Industry (Tokyo, Japan). The bLF preparation used in this study contained iron (0.019% w/w) and the saturation was 14%. LFcinB was purified from bLF pepsin hydrolysate, as previously described [46]. Recombinant human COMT was purchased from ATGen (Seongman, South Korea). Entacapone was purchased from Novartis International AG (Tokyo, Japan). Casein Na was purchased from Wako Pure Chemical Industries (Tokyo, Japan). Perchloric acid (70% solution) was purchased from Kokusan chemical (Tokyo, Japan). ^14^C-labeled *S*-adenosylmethionine (SAM) was purchased from PerkinElmer (Yokohama, Japan). Other chemicals were purchased from Sigma-Aldrich (Tokyo, Japan). The amino acid sequences of polypeptides P4 and P5 were designed from the sequence of bLF and synthesized by PH Japan (Hiroshima, Japan). The amino acid sequences were as follows: P4, ALECIRAIAEKKADAVTLDGGMVFEAGRDPYKLRPVAAEIYGT and P5, VRWCTISQPEWFKCRRWQWRMKKLGAPSITCVRRAFALECIRAIA. The P5 peptide has two disulfide bonds (Figure 1C). 

### 4.2. Preparation of bLF Fragments p36, p20, and p51 by Trypsin Digestion

bLF fragments P20, P36, and P51 were prepared by trypsin digestion [47,48]. The reaction mixture was comprised of 75 mg of bLF in 7.5 mL of 50 mM Tris-Cl (pH 8) containing 10 mM CaCl_2_ and 2 μg/mL trypsin (Wako Pure Chemical Industries, Tokyo, Japan). The reaction mixture was incubated at 37 °C for 24 h. The peptides were then separated using a reverse-phase HPLC system; Phenyl-5PWRP (75 × 4.6 mm, Tosoh, Tokyo, Japan), 0–80% acetonitrile gradient containing 0.5% heptafluorobutyric acid, 0.8 mL/min. Peak fractions were further purified using a reverse-phase HPLC system; Phenyl-5PWRP (75 × 4.6 mm), 5–65% acetonitrile gradient containing 0.1% trifluoroacetic acid. The fractions were then dried, dissolved with H_2_O, and assayed for COMT activity. Molecular weights of these fragments were estimated to be 36, 20, and 51 kDa, by following previously published methods [43,44]. The *N*-terminal sequences of these fragments were APRKN (P36), ESPQT (P20), and SFQLF (P51). The molecular weights of peptides are; P5, 5 kDa; P4, 4 kDa; and LFcinB, 3 kDa.

### 4.3. COMT Assay

COMT activity was determined as described by Floderus et al. [49]. Briefly, a typical reaction mixture (25 µL) was composed of 0.004 mg/mL COMT protein, 50 mM Tris-HCl (pH 8), 2 mM MgCl_2_, 11.2 μM ^14^C-labeled SAM, 1 mg/mL bovine serum albumin (BSA), 1 mM DTT, inhibitors (bLF, bLF-derived peptides, entacapone), and 0.5 mM dihydroxybenzoic acid (DBA). Radioactivity was ascertained to be approximately 21,000 disintegrations per minute per reaction tube. The reaction was initiated by the addition of 2.5 μL DBA (5 mM; H_2_O solution) and reaction mixtures were incubated at 37 °C for 10 min. The reaction was stopped by the addition of 12.5 μL HCl (1 M). A 300 μL mixture containing toluene and isoamyl alcohol (7:3 *v/v*) was added to the reaction mixture and vigorously vortexed for 20 s. After brief centrifugation, the upper phase (200 µL) was recovered and its radioactivity was measured using a scintillation counter. Reaction mixtures contained 1 mg/mL BSA to prevent COMT from non-specific absorption to the reaction tube. 

### 4.4. Inhibitory Activity of bLF against DTT-Pretreated COMT

To a mixture of recombinant COMT (0.0049 mg/mL), 61 mM Tris-Cl (pH 8), 2.4 mM MgCl_2_, 13.7 μM ^14^C-labeled SAM, and 1.2 mg/mL BSA cooled to ice cold, a DTT aqueous solution was added to give a final concentration of 1.2 mM. The mixture was dispensed into portions of 20.5 µL at 0 °C. Each dispensed reaction solution was incubated for 0, 5, 15, 30, and 60 min at 37 °C and was cooled on ice for more than 10 min. Then, 2 μL bLF (67 µM) or P5 (8 µM) was added at 0 °C. Each reaction solution was incubated at 37 °C for 15 min followed by the addition of 2.5 μL of 5 mM DBA to start the enzyme reaction. The control mixture did not contain bLF or P5. The experimental scheme is shown in Figure S4.

### 4.5. Cell Culture and Preparation of Cell Lysates

Human liver cancer (HepG2) and epithelial colorectal carcinoma (Caco-2) cell lines were purchased from DS Pharma Biomedical (Suita, Japan). HepG2 and Caco-2 cells were cultured in a 12-well plate. HepG2 cells were cultured in Dulbecco's Modified Eagle's medium (DMEM) containing 10% bovine serum and were cultured until 100% confluence. Caco-2 cells were cultured until 100% confluence in DMEM containing 10% bovine serum and 2 mM of glutamine. In order to facilitate the differentiation of Caco-2 cells, 2–5 mM of sodium butyrate was added and the cell culture was maintained for 2–3 weeks. Cultured cells were washed with PBS twice and suspended in 200 μL PBS (−DTT lysate) or PBS containing 5 mM DTT (+DTT lysate). Suspensions were frozen and kept at −80 °C for a day. Cell lysates were prepared using the freeze and thaw method. Frozen cells were thawed on ice and gently vortexed. They were then centrifuged at 15,100× g for 15 min for the preparation of cell lysates. For assessing COMT activity of cell lysates, the buffer component of the enzyme reaction mixture was 50 mM phosphate buffer (pH 7.8) instead of Tris buffer. COMT activity (−DTT lysate) was measured in the presence or absence of 1 mM DTT. Lysates were added to the reaction mixture and incubated for 15 min at 37 °C. Addition of DBA started the reaction and the incubation was continued at 37 °C for 15 min. For the experiment assessing the concentration dependency of bLF and P5 on the inhibition of COMT reaction, DTT was omitted from the reaction mixture. The protein concentration in cell lysates was determined by the Bradford method using a kit provided by Bio-Rad (Tokyo, Japan).

### 4.6. Immunoblotting

The cell lysates of HepG2 and Caco-2 cells (15–22 μg of protein) were subjected to SDS-PAGE (12% gel). The proteins were then transferred on to a PVDF membrane, which was blocked with skim milk. Immunoblotting was performed with the ECL system (GE Healthcare, Japan). The first antibody used was rabbit polyclonal COMT antibody (GeneTex, Inc., Irvine, CA, USA; Catalog No. GTX101233). For detection of chemiluminescence signal, ChemiDoc XRS+ (Bio-Rad, Japan) was used.

## Supplementary Materials

Supplementary materials are available online: Table S1 and Figures S1–S4.
