# Supplementary material for: Inhibitory Effect of Bovine Lactoferrin on Catechol-O-Methyltransferase
Source: Molecules. 2017 Aug 19;22(8):1373. doi: 10.3390/molecules22081373 (PMC6152271; doi:10.3390/molecules22081373)
Supplement: Supplementary file 1 [file molecules-22-01373-s001.pdf]

# Supplementary Materials: Inhibitory Effect of Bovine Lactoferrin on Catechol-*O*-Methyltransferase

**Masayuki Ikeda *, Hiroshi Iijima , Ichizo Shinoda , Hiroshi Iwamoto , and Yasuhiro Takeda**

**Table S1.** Inhibitory effects of bovine proteins to COMT activity.

| **Protein** | **IC50** | |
|---|---|---|
| | **mg/mL** | **μM** |
| Bovine Lactoferrin | 0.13 | 1.6 |
| Casein Na | >5.8 | ND |
| βLactoglobulin | >10 | >540 |
| BSA (Bovine serum albumin) | >17 | >260 |
| αLactalbumin | 6.7 | 480 |
| γGlobulin | 4.1 | ND |
| Apo-Transferrin | >8.7 | >110 |
| Entacapone | $0.024 \times 10^{-3}$ | 0.079 |

Molecular wighits were: Bobine Lactoferrin, 80 kDa; βLactoglobulin, 18.4 kDa; BSA, 66 kDa; αLactalbumin, 14 kDa; Apo-Transferrin, 80 kDa; Entacapone, 305 Da. ND: not determined. Molarites of γ-globulin and casein Na were not estimated because they are heterogeneous mixture of various molecules.

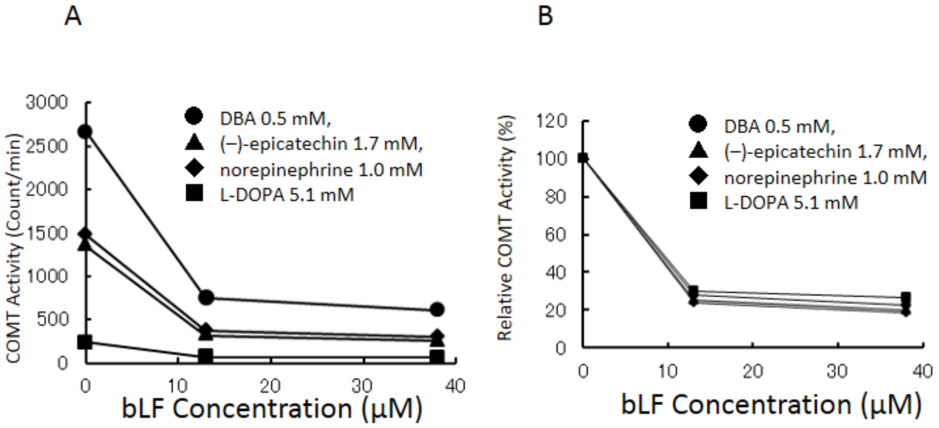


**Figure S1.** (**A**) Inhibitory activity of bLF on COMT reaction with various substrates. bLF (0 μM, 13 μM, and 38 μM) was incubated with COMT and various methyl accepting substrates. A. The vertical axis represents the amount of methyl transfer shown as scintillation count. (**B**) The same data set shown in the panel A was replotted in terms of the relative COMT activity. The relative activity was calculated base on the amount of the methyl transfer in the absence of bLF for respective substrates. When the methyl acceptor substrate was norepinephrine or L-DOPA, the reactions were stopped by the addition of 0.5 M boric acid. In the case of norepinephrine and L-DOPA, isoamyl alcohol was used to extract the reaction products.

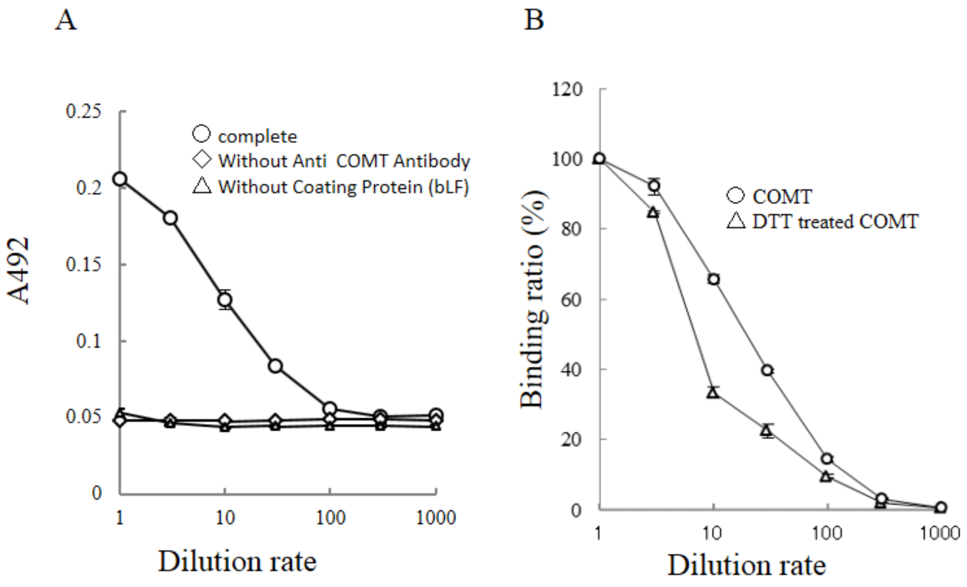


**Figure S2.** Binding of bLF and COMT assessed by ELISA. (**A**) Binding of COMT which was not treated with DTT is shown. The vertical axis represents the reading of absorbance from ELISA experiments. Some wells that were not coated with bLF and some to which anti-COMT antibody was not added were prepared for the control (background) measurement. Reading of the absorbance from these wells are also indicated as control plots. (**B**) This panel shows the difference in the binding affinity of COMT preparations. The COMT preparation treated with DTT before the binding experiments indicated lower binding affinity to bLF than non-treated preparation. This ELISA experiment was repeated twice independently and values are described as average and SD. Method: The ELISA plate was coated with 20 μg/mL of bLF and incubated at 4 ℃ for 18 h, and then washed with phosphate-buffered saline (PBS) and blocked with 1% BSA at RT for 2 h. After washing with PBS, COMT solutions were added to the wells. The COMT stock solution (10 μg/mL) was diluted 3-, 10-, 30-, 100-, 300-, and 1,000-fold with PBS. After 1 h of incubation at RT, the wells were washed with tween-PBS and anti-COMT antibodies were added (Everest Biotech Ltd., UK; Catalog No. EB06595), followed by 1 h of incubation at RT and washed with tween-PBS. The secondary antibody (peroxidase-labeled anti-goat IgG) was added, incubated at RT for 1 h, then wells were washed with tween-PBS, and the coloring reaction was performed using *o*-phenylenediamine. Biding ratio of COMT to the fixed bLF was described in percentage, which was calculated as relative ratio of the reading to the non-diluted specimen.

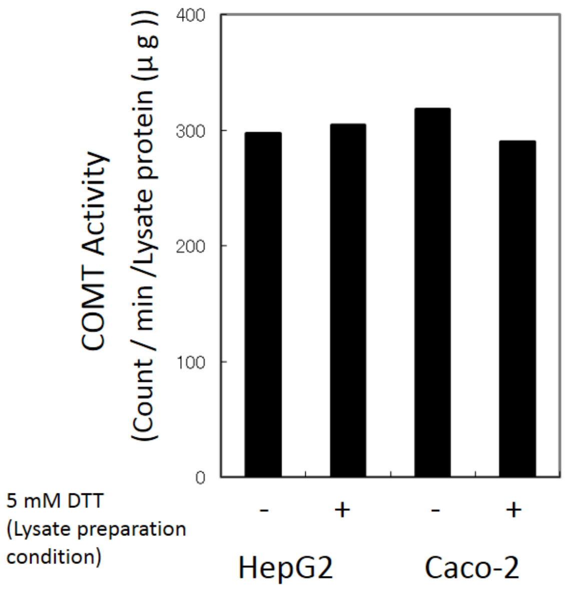


**Figure S3.** COMT activity of cell lysates extracted in the presence of 5mM DTT or in the absence of DTT. The reaction mixture of COMT assay (25 μL) was composed of cell lysate (HepG2 or Caco-2), 50 mM phophate-buffer (pH 7.8), 2 mM $MgCl_2$, 11.2 μM $^{14}C$-labeled SAM, 1 mg/mL BSA, and 0.5 mM dihydroxybenzoic acid (DBA). Reaction carried out without DTT at 37℃ for 15min. COMT activity was corrected with amount of lysate protein in reaction mixture.

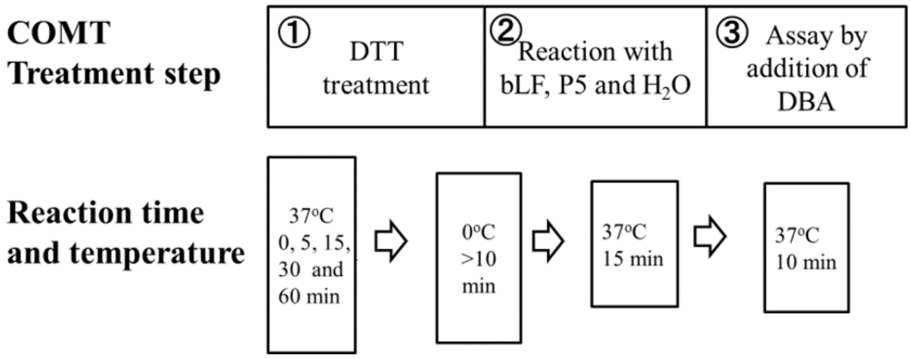


**Figure S4.** Experimental Scheme of Figure 3. Inhibitory activity of bLF against DTT-pretreated COMT
